# Intent to leave academia: perceptions and challenges of nurse faculty

**DOI:** 10.1186/s12912-024-02137-y

**Published:** 2024-07-27

**Authors:** Nouf Afit Aldhafeeri, Fadiyah Jadid Alanazi

**Affiliations:** 1 College of Nursing, King Saud bin Abdulaziz University for Health Sciences, Riyadh, Saudi Arabia; 2 King Abdullah International Medical Research Center, Riyadh, Saudi Arabia; 3 Ministry of the National Guard - Health Affairs, Riyadh, Saudi Arabia; 4 Public Health Department, College of Nursing, Northern Border University, Arar, Saudi Arabia

**Keywords:** Nursing, Faculty, Intent to leave, Academia, Challenges, Perception

## Abstract

**Background:**

The shortage of nursing faculty is a significant global issue affecting the nursing profession. Faculty turnover can negatively impact the institution by decreasing the number of qualified nursing faculty and disrupting the educational process. The cost of replacing departing faculty and recruiting and training new faculty may increase.

**Purpose:**

To describe perceptions of nursing faculty of intentions to leave academia and to identify challenges that contribute to nurse faculty turnover in academic setting.

**Method:**

A descriptive naturalistic, qualitative approach is used to explore nurses perceptions and intentions to leave academia.

**Results:**

The themes identified were unexpected journey, with two sub-themes, lack of role clarity and lack of preparation/orientation, and work environment, with four sub-themes: low Salary, workload, lack of support and favoritism.

**Conclusion:**

The urgency of addressing the factors that contribute to nurse faculty intending to leave academia cannot be overstated. The results of this study have direct implications for nursing schools’ administrators, where these findings can provide them with, valuable insights that they can be used to implement best practices and mitigate the problem of nursing faculty turnover.

**Supplementary Information:**

The online version contains supplementary material available at 10.1186/s12912-024-02137-y.

## Background

The nurse faculty shortage is a significant and increasing concern in academic nursing communities with far-reaching implications for the nursing profession [1]. Nursing students’ educational and clinical requirements are inadequately addressed due to a shortage of qualified nursing faculty [1]. Critical problems within nursing academia revolve on the intent of nurse faculty members to withdraw from academia and the barriers they encounter. Nurse faculty play a central role in shaping the future of nursing by educating and mentoring the next generation of healthcare professionals. When qualified professionals leave academia, this leads to a significant deterioration in the quality of nursing education and poses a threat to the healthcare system. The departure of specialists from the academic field therefore means that new teaching staff must be trained and the workload for the remaining teaching staff increases.

Comprehending the underlying causes of the nurse faculty shortcoming will facilitate the development of mitigation strategies. Implementing research-based strategies, including supportive work environments, improved management practices, and competitive salaries, can help mitigate nursing faculty shortages, attract and retain skilled educators, and safeguard the nursing profession.

Recent studies have shown that more nurses are expressing their intention to leave the academic world [2–4]. Furthermore, a comprehensive investigation has been conducted to reveal factors that lead to the departure of nursing faculty members from academic institutions.

A 2017 Ethiopian study revealed that an unhealthy work environment, insufficient facilities, poor management, and dissatisfaction with salaries are the main contributing factors for faculty members to leave academia. Out of 217 faculty members, 164 expressed their intention to leave, with 71.3% identifying this as a critical issue. Inadequate basic amenities and compensation also played a significant role in their decision to leave the academic community [2].

A Finnish study in Finland found that 50% of 3,760 participants expressed turnover intention, primarily due to lack of professional commitment, dissatisfaction with the school system, and heavy workload. Addressing these issues is crucial as faculty turnover can negatively impact institutional progress. Investigating these issues can help create a healthy working environment and promote faculty retention [3]. Ulmen and Lloyd found that 23.3% of nursing faculty experienced burnout and compassion fatigue, negatively affecting their intent to stay in academia [4]. Factors such as high workload [5], poor work quality [6], unsupported environment [7, 8], job dissatisfaction, and low commitment [8] contribute to faculty intent to leave academia. Addressing these predictors can help improve the work environment, reduce burnout, and enhance faculty satisfaction, retaining experienced faculty and ensuring quality education.

A study conducted by Roughton on the intentions of nursing faculty to leave identified several critical factors that influenced their decision, including retirement, workload, salary, career development opportunities, performance recognition, and work-life balance. These factors were all influenced by the six-domain model of the healthcare system, regulatory environment, financing system, education system, technology, and work environment [10]. Other factors that contribute to the retention of nurses include job satisfaction, education level, and age [11].

An alarming decline has been seen in the number of qualified nursing faculty members in the US. In 2019, the American Association of Colleges of Nursing (AACN) reported a faculty vacancy rate of 7.2% [12]. This vacancy rate denotes the proportion of faculty positions that remain vacant in nursing schools throughout the US. The academic milieu, salary, incivility, excessive burden, and faculty aging are among the numerous factors that contribute to lower faculty retention, as documented in the literature [13–15]. It is evident from the literature review that, even though a significant amount of research is dedicated to strategies for faculty retention, there is a need for additional research to concentrate on the retention of current faculty.

Faculty retention can begin with solid academic leadership within the institution and individual schools/colleges of nursing. Research shows that support and empowerment from deans and other leaders plays a critical role in improving faculty job satisfaction and fostering their intent to remain in their faculty role [16, 17]. Mentoring for new faculty members is an effective strategy to increase faculty retention [18, 19]. By providing guidance, support, and professional development opportunities, mentoring programs can help new faculty members navigate the challenges of their role and build a solid foundation for their academic careers. Mentoring can build confidence, foster teamwork, and enable the growth of new nurse faculty. The relationship between mentor and mentee promotes greater job satisfaction, making it an effective tool for faculty retention [18, 19].

Most research on faculty retention and intent to leave academia has relied primarily on quantitative methods. Aquino et al. conducted a quantitative study to explore burnout and intent to leave among nursing faculty in the US [20]. In particular, the study examined the differences between faculty with a PhD and those with a DNP degree. Participants considered novice faculty with five years or less of academic teaching experience experienced higher levels of stress, exhaustion, and burnout, possibly due to the demands associated with tenure-track requirements. However, the authors pointed out that a limitation of this study is that it did not include qualitative data collection. Therefore, to gain a deeper understanding of burnout in academia and the reasons for wanting to leave academia, it would be valuable to conduct interviews with participants and understand their feelings in depth. Such qualitative findings could shed further light on the experiences and motivations related to burnout and faculty turnover.

Turnover rates among nursing faculty pose challenges to maintaining a stable workforce, potentially disrupting nursing education and affecting healthcare delivery quality. Further qualitative research is needed to understand faculty perceptions of academia and their intentions to leave their positions in Saudi Arabia. This will contribute to nursing knowledge and help nursing administrators develop effective strategies to address this issue. Further studies are needed to understand faculty perceptions and develop effective strategies.

### Significance of the study to nursing administration

The study is relevant to nursing administration, particularly in Saudi Arabia, as it provides insight into the views of nursing faculty who intend to leave academia. Faculty turnover has a significant impact on nursing education as it can affect student performance and reduce revenue for nursing schools. Therefore, nursing administrators need to explore and address the factors that contribute to faculty intentions to leave academia in order to address the nursing faculty shortage.

The results of this study are promising and can provide valuable insight to nursing schools’ administrators. By understanding the perceptions of nursing faculty regarding their intent to stay in or leave academia, administrators can identify areas for further improvement and development to improve faculty retention. The results of this study serve as a promising foundation for best practices and a guide for further implementation of effective interventions to support faculty members and encourage their retention to remain in their academic roles. Nurse leadership can help create a stable and supportive environment that foster faculty satisfaction and engagement to ensure quality education for nursing students and contribute to a sustainable nursing workforce.

The aim of this study was to describe how nursing faculty members perceive their intention to leave academia. The second objective was to describe perceptions of academic challenges that contribute to nursing faculty leaving academia.

Operational definition:

For the purpose of this study, the term nursing faculty member refers to any faculty member who holds an academic rank or position or classification and teaches courses in nursing, both theoretical and clinical.

## Method

This study was conducted among nursing faculty in Saudi Arabia. The researchers used social media platforms (WhatsApp) to reach the participants who met the inclusion criteria for the study. In this social platform, there is a group of nurse faculties from all nursing colleges. A message with a link to the screening questions was sent to the group. All faculty members who were eligible were contacted personally to arrange a meeting. Interviews were conducted using semi-structured interview questions [Appendix 1] to gather in-depth knowledge and allow participants to express their feelings, challenges, and reasons for leaving academia. The interview questions were formulated based on the literature review, focusing on participants’ unique perspectives. This approach allows for a comprehensive understanding of participants’ perspectives and experiences, making their voices an integral part of the research. Participant interviews were planned and arranged in collaborative between the researchers and the participants. Interviews were digitally recorded via the Zoom platform to ensure that no valuable information was lost in the analysis. All recorded interviews were transcribed verbatim. Participants were asked to keep the video camera open during the interview to establish a good rapport with the participants and to easily record non-verbal reactions.

### Study subjects

The inclusion criteria were nurse faculty currently teaching in Saudi Arabian nursing schools who were thinking of leaving academia based on the screening question, who had been employed as a nursing faculty for at least one year member, and who could communicate in English, as English is the acceptable language in all nursing programs. We excluded faculty who have employed in nursing for less than one year and faculty who are not employed in nursing and who cannot communicate in English.

### Study design

The researchers used a descriptive qualitative design that supports a naturalistic approach to understand the perceptions of nursing faculty regarding their intention to leave academia in Saudi Arabia. This study approach captures the unique perspectives and underlying motivations that influences nurses’ decision to leave academia. The naturist approach allows the researcher to maintain the authenticity of the data and present the participants’ experiences without preconceived notions or manipulation of the study variables [21]. Consequently, this study provided a comprehensive understanding of the experiences of nurses intending to leave academia, their challenges in the broader context of their socialization, and the various influences on their academic role from a holistic perspective.

### Sample size

This study reached data saturation with eight participants. The purposeful selection of respondents allowed for an in-depth understanding of a phenomenon. After the five participants, the data was repeated with the same information, and the authors determined that the data was saturated at eight participants.

### Sampling technique

In order to recruit participants and comprehensively represent the target participants, a purposive sample was drawn. This type of sampling would help the researcher to search for participants who met the inclusion criteria. This sampling procedure helped to obtain rich information about faculty leaving academia and the challenges that formed the themes of the study.

## Data collection and instruments used

Data collection begun after IRB approval was obtained. The researchers used social media to recruit participants (WhatsApp), briefly describing the purpose of the study and providing a link to screen participants, in which they answered two questions and provided the researchers with their contact information. Over the course of three months, data was gathered through one-on-one online interviews, with the utmost respect for the privacy and time of the participants. A professional transcriptionist transcribed the digital audio recordings verbatim and reviewed them for accuracy. To ensure complete comprehension, nonverbal indicators, including body language, were observed, and documented as necessary. Our data had additional enhancements and bias was eliminated by taking notes during the interview.

Demographic information and an informed consent form were obtained from each participant prior to the interview. Zoom was employed to conduct the individual interviews, which was favorable for the participants. The estimated time for each interview was 30 to 45 min. Interviews were recorded via Zoom so that they could for later be transcribed verbatim coded. The interview questions began with general, open-ended questions to build rapport and trust with the participants, followed by questions related to targeting the participants’ beliefs about leaving academia. Then, additional probing questions were asked based on the participants’ responses. The interview procedure was designed to gather data until saturation was reached.

## Data management and analysis plan

After the data had been collected and transcribed by a professional transcriptionist, the researchers checked the transcription for accuracy. The data obtained from the transcriptions were then analyzed using a five-step process described by Barritt et al. [22]. This process was designed to systematically analyze qualitative data and identify significant themes and patterns within the data. The researchers read the transcript and identified significant elements, statements, or ideas that emerged from the interview data. The researchers then began to group similar ideas or concepts and assigned preliminary themes to these groupings. To ensure the accuracy and validity of the analysis, researchers solicited input and clarification from participants, which can add depth to the analysis. The researchers compared the narratives of different participants to identify common themes that emerged across many interviews and to find unique themes. Finally, the researchers examined the literature on nurse faculty intending to leave.

## Study rigor

Lincoln and Guba’s four criteria of credibility, dependability, confirmability, and transferability were implemented to ensure the study’s rigor [24]. The credibility of the findings was established through member checks. These checks involved sharing transcript summaries and initial interpretations with participants, seeking feedback and validation, and ensuring accurate representation of participants’ perspectives. Participants with experience who intended to leave academia were chosen to maintain trustworthiness and data rigor. The dependability of the data was enhanced by following the audit trail and field notes and storing all reflections throughout the audit. The data were reviewed and confirmed to ensure that they matched with the participants’ responses. To further enhance the confirmability of the findings, two expert researchers verified the accuracy of the data analysis. Personal biases and assumptions were documented in journals. To ensure the transferability of the findings, participants from various nationalities, regions, years of experience, and positions were included in this study. Member-checking, peer debriefing, audit trials, and reflexivity were used to verify data accuracy [25].

## Confidentiality

The researchers ensured that any involvement in the study was entirely voluntary. Researchers protected the privacy of the participants by using numerical identifiers instead of their actual names. The researchers confirmed that no information about the university would be recorded or shared. All research-related materials, such as notes, transcripts, consent forms, reflection journals, and audio recordings, were maintained securely stored on a computer that require password for access. Only approved researchers were able to access this computer. To prevent any unintended interruptions, the researcher used a waiting room feature during the Zoom interview session.

## Findings

### Characteristics of participants

The researchers invited 83 participants from different Saudi nursing schools. Only eight participants responded to the screening tool; however, data saturation was achieved. Interviews lasted between 30 and 45 min (mean = 35 min). Participants were Saudi and non-Saudi faculty members. Participant’s data included two faculty members from northern Saudi Arabia, two from western Saudi Arabia, one from central Saudi Arabia, and one from eastern Saudi Arabia. Most of the sample was female (*n* = 7). One participant was from a private school and the rest were from public schools (Table 1).

**Table 1:** Demographics of the participant

Demographics	*n*	%
**Gender**		
Male	1	12.5
Female	7	87.5
Age M = 38.4		
**Marital status**		
Married	7	87.5
Single	1	12.5
**Education background**		
Master	3	37.5
Ph.D.	4	62.5
**Years of experience**		
1–2 years	0	
3–5 years	5	62.5
6–8 years	0	
9–11 years	1	12.5
More than 11 years	2	25
**Nationality**		
Saudi	6	75
Non-Saudi	2	25

The process of data analysis was meticulously conducted and resulted in the generation of two main themes that answered the research questions comprehensively.

### Unexpected journey

The theme “Unexpected journey” with two sub-themes, namely lack of role clarity and lack of preparation, answered the main research question, “: What are the nursing faculty’s perceptions of intent to leave academia? (Table 2)”.

**Table 2:** The major themes and subthemes

Major category themes	Subthemes
1. Unexpected journey	1.1 Lack of role clarity
	1.2 Lack of orientation/preparation
2. Work environment	2.1 Low Payment
	2.2 Workload
	2.3 Lack of support
	2.4 Favoritism

#### Lack of role clarity

The initial perception of the faculty was exhaustion from working in the academic environment. Although the faculty members wanted to be in academia because of their passion as educators and their belief that they are good educators, they were surprised after almost 3 to 4 years of experience. They thought about leaving the academic world. The majority of faculty members were taken aback by the demands of academia and did not anticipate that they would consider departing. One participant said, “*I thought I would come to teach some materials. Teach some components. Give some lectures, and that is it. However, the academia is mixed between teaching, documenting, and doing administrative duties*.” In addition, participants pointed to role ambiguity, a term used to describe the lack of clarity or understanding about one’s responsibilities and expectations in a particular role. Participants mentioned, ”*All we need is an organization for the rules of academic staff from the beginning.”* They also stated, *“I thought it would be organized and easy to work, and I would have at least a job description; however, I felt I liked working in a bizarre environment. For example, I was often unsure about whether I was expected to focus more on research or teaching, and this lack of clarity made it difficult for me to prioritize my tasks.”*

#### Lack of preparation/orientation

Faculty are concerned that once they start working in academia, they have no preparation to ease their transition. One participant said, *“It was not clear to me the role of the faculty at that time, I found there were some management sides not only teaching ………, you know, paper quality work, and I thought it was maybe a piece of cake, compared to the clinical site. It might be more accessible. I thought so*.”

Other participants said, *“I have three years now. I did not see myself involved in academia yet, not at all.”* Another participant mentioned that “*they expect us to do everything at the beginning. Without orientation, for example, the quality of work. This is a big commitment and has too many details. They let me be the head of the quality committee without knowing anything. I was stressed about how to meet their expectations or goals.”* The participants felt that *“the organization…. need to prepare new faculty for the work environment and/or the roles of the academic staff.”*

Following their completion of their degrees abroad, some Saudi Arabian faculty members returned to their respective institutions with a fervor for their professions. Nevertheless, they are considering leaving from academia after encountering the workload without adequate preparation.

### Work environment

The second research question “What challenges in academia contribute to nurse leaving academia?”, addressed the theme of work environment and its sub-themes, including low salaries, workload, lack of support and favoritism. Most participants expressed dissatisfaction with the working environment in academia.

#### Low salaries

Several individuals expressed discontent with their current pay and were actively seeking other alternatives, such as employment in the private sector or with other firms.

One participant explained, “*Comparing to the salaries that I would receive, it is very compelling for me…to have an appealing offer. Why not? Why would I stay in a place where I’m feeling stressed out when I’m feeling left out, left behind, ignored, overlooked, and disrespected? Not appreciate it. At the same time when I can go somewhere else and get more respect*.” Another participant mentioned that “*the salaries are not equivalent to the stress and the effort that we spend, and the time that we spend working on many things.”* A female faculty member contemplates the prospect of venturing into areas that need a female workforce to fulfill the goal of promoting female empowerment by 2030. Participant 5 said, *“I am thinking about going to companies or something in business, private sector because they have many chances, or on top of opportunities because they are looking for a female. They have the positions.”*

#### Workload

The majority of the participants emphasized that nursing faculty have a lot to do. Participants noted that they have to take care of quality, teaching, administrative work and the development of original research. One participant explained “….*stress because of the workload. We have an extra teaching load. We have administration loads. So even if I am not in an administration position, I need to work in the administration work*.” Another participant stated, “*I teach up to fourteen credits per semester. I have no time*”. Other participants expressed that the workload “ *keeps me away from teaching students as well as conducting research and community services.*” In addition, participants were also unsatisfied with the distribution of work and committees among staff. They recognized that the workload came from the administrative staff and the head of department because they “dumping the responsibilities onto you.” Another participant stated that “*they let me be the head of the quality committee without knowing anything*.”

#### Lack of support

When we asked participants about the factors that affected their feelings about leaving academia, almost all participants agreed that there was a lack of support in addition to the heavy workload. One participant mentioned that *“ we need motivation. Even if we have Ph.D., we need support from the administration in terms of career guidance and mentorship.”* Another participant explained: *“we need support not only financial but can be research support and allowances such as funding for conferences and research materials.”* Participants needed the support to be close to them and to understand their feelings. Participants mentioned that “*the support is important, and we also need to have people who believe these are things, or situations need to be changed…you need to be close to the faculties and understand their issues*.”

Other participants added that the allowance and promotion are rigorous, which limits motivation to stay academically. “*The promotion process is complicated, and the allowances they did not give you all the time you have to complete your credit hours and apply for that every semester.”* Participants felt that they needed support and appreciation for their work. One participant said, “*The lack of appreciation, or even sometimes respect, sometimes of the managers they do not even have appreciation or respect to what you are doing*.” Participants emphasized the need to be involved in decision making to support them and to ensure that the faculty are satisfied and working effectively. One participant said: *“Shared a decision making is important,……. it will support us….”*

#### Favoritism

Some participants expressed concern about favoritism in the work environment. For example, when some managers do not believe in the abilities of faculty members, they do not assign them work and allow other faculty members to serve on many committees. This has led to a sense of marginalization and demotivation among faculty members. One participant stated, “*Some people will have some favorite group of people; you will see these people if I would say the higher administration*.*”* Another participant said that “*having them as best will be involved in everything, and others will be excluded.”* These issues not only affect faculty morale but can but also affect the quality of work and overall productivity.

## Discussion

Saudi and non-Saudi faculty members teach nursing students at Saudi Universities. The nurse faculty shortage is a global issue; administrators need to retain them and increase faculty retention. Investigating the reasons for faculty attrition intentions will help find a solution to the faculty shortage problem. Two main themes emerged from the data analysis: an unexpected journey with sub-themes of lack of role clarity and lack of orientation/preparation; the second theme was work environment with sub-themes of low salaries, workload, lack of support and favoritism. These findings emphasize the need for improved role clarity, better orientation and preparation for new faculty, and a supportive work environment to increase faculty retention and address the issue of nurse faculty shortage.

### Unexpected journey

The theme of the unexpected journey related to the transition from clinical practice to academia explains the sense of shock of the participant who did not expect this journey. Overall, the participants was ready to work and excited to put all their effort into the academic world. However, after years of experience, they were faced with an unexpected journey. This theme relates more to faculty with one to three years of experience who are considered new faculty. Several research studies have found similar experiences to this current study that examined the experiences of nursing faculty [11, 26, 27]. Study participants explained their journey as frustrated and unprepared for their academic work and needed a clearer understanding of their role as faculty members.

### Lack of role clarity

The sub-theme represents the participants’ feeling that their role is unclear, that they are struggling and that their work is frustrating. The faculty role is different from the clinical and practicum role. Therefore, the transition from a practitioner role to an academic role caused a lack of role clarity. This finding is similar to several studies looking at nurses’ experiences in an academic setting in which a lack of role clarity has a negative impact on faculty members [26–28]. Participants in this study stated that they felt lost when they did not have a clear understanding of their academic responsibilities and expected role. Similar results were found [27, 31] where first year faculty members experience role uncertainty and deal with a lack of self-confidence. These previous studies supported our current finding where faculty thought their role was only teaching and they experienced a lack of clear role descriptions [31]. These findings have significant implications for nursing education and practice as they point to the need for clearer role definitions and support for faculty moving from clinical to academic roles.

### The lack of orientation/preparation

The participants in this study felt compelled to leave academia when they experienced a lack of orientation and preparation for work in academia. Most nursing degree programs prepare students for clinical work in the hospital, not for faculty work in academia. Therefore, participants did not feel prepared for teaching work, which increased their willingness to leave academia. The findings of the current study are similar to those of several studies in which new faculty struggle with a lack of orientation and preparation for academic roles [26, 28, 32–34]. One study has shown that preparing faculty members for their jobs increases their intention to stay in academia [19]. The fact is that most Bachelor of Science Nursing (BSN) degree programs prepare students clinically, rather than theoretically, to work in an academic setting, which can lead to a stressful transition into academia and potentially leaving the profession [35, 36]. Many researchers support the idea that orientation programs and preparing faculty for the academic.

environment can significantly increase satisfaction and help retain faculty in academia [37, 38].

### Work environment

The topic of work environment was comprehended within the framework of inadequate compensation, excessive workload, insufficient assistance, and favoritism. The work environment was a contributing factor in the participants’ decision to abandon academia, as they emphasized in this study.

### Low salaries

Several participants stated that they were dissatisfied with their current salaries. These findings were consistent with previous studies [2, 10]. Ibrahim et al., highlighting the pervasive problem of low salaries in practice. Ibrahim et al., found that more than half of faculty members are dissatisfied with their salaries [2]. Roughton found that faculty members reported that they receive inadequate salaries [10]. This situation is not unique to Saudi Arabia and could lead nursing faculty members to leave academia and seek outside opportunities where they can receive higher salaries.

### Workload

All participants agreed that the heavy teaching and non-teaching workload influences their intention to leave. Workload is one of the biggest factors that challenge nursing faculty and cause them to consider leaving academia. This finding is similar to the results of previous studies [3, 5, 10]. Bettini et al. mentioned that prospective educators who reported that their work is unmanageable are emotionally exhausted, which leads them to consider leaving their job [5]. Similarly, Räsänen et al. found that the main factors were high workload and unequal distribution of work between teachers [3]. Roughton mentioned that nursing teachers believe that their workload is higher than that of non-nursing teachers, which is increased due to the shortage of teachers [10]. Thus, the workload could cause nursing faculty members to leave academia.

### Lack of support

Most participants agreed that lack support and appreciation would influence their intention to leave academia. These findings are consistent with the results of some studies [2, 10, 39]. Yedidia et al. described that nursing faculty were dissatisfied with the availability of administrative support in their school and the relationship with their school administration, which contributed to their intention to leave [39]. In addition, faculty were not satisfied with the availability of professional development and rewards. Roughton and Ibrahim et al. found that lack of recognition for performance was a major reason for leaving [10, 2].

### Favoritism

This is another new finding of this study. Participants expressed that some of their managers favored other faculty members. Participants felt that this was because they wanted to get the work done quickly or that these faculty members supported their decisions even though they knew they were wrong. Favoritism in academia can have a negative impact on individuals, leading faculty to consider leaving academia. Favoritism can cause to unfair treatment of staff and create an environment where certain individuals or groups are given preferential treatment while others are overlooked or disadvantaged. Studies show that favoritism in the work environment can cause to employees’ disengagement [40–42]. Favoritism can be seen as incivility on the part of the supervisor and undermines the principles of fairness and meritocracy that are critical to maintaining the integrity of academia [40, 41]. When faculty that they were members feel that favoritism is at play, it can reduce motivation. Faculty who felt overlooked despite their qualifications and efforts lost their enthusiasm for the work and may have been inclined to leave academia.

### Implication for nursing administration and nursing leadership

Nursing faculty members’ intention to leave school can affect student performance and reduce nursing schools’ revenues. In order to reduce the nursing faculty shortage, strategies need to be developed to investigate and manage intent to leave. The knowledge gained from this study can help nursing administrators develop plans to retain, mentor, and support nursing faculty. The nursing administrator must understand the needs of faculty members in order to prepare them and create a supportive environment that fosters their confidence and teaching ability.

## Conclusion

The findings of this study address the perceptions and challenges of dealing with the intention to leave among nursing faculty members. Addressing the factors of intent to leave factors may improve faculty retention, satisfaction, and performance, which could impact student performance and increase revenue for schools of nursing. The study has some limitations in its recruitment process regarding the use of social media for recruitment. This procedure could limits access to participants who were not active in social media.

### Electronic supplementary material

Below is the link to the electronic supplementary material.

Supplementary Material 1

## Data Availability

Due to the participants confidentiality, data are available from the corresponding author upon reasonable request.
