# Supplementary material for: Intent to leave academia: perceptions and challenges of nurse faculty
Source: BMC Nurs. 2024 Jul 27;23:506. doi: 10.1186/s12912-024-02137-y (PMC11283686; doi:10.1186/s12912-024-02137-y)
Supplement: Supplementary file 1 — Supplementary Material 1 [file 12912_2024_2137_MOESM1_ESM.docx]

**Appendices:**

***Screening Question:***

| 1.            Are you a nursing faculty teaching at a nursing school? |
| --- |
| Yes         No |
| 2.            Have you thought of leaving academia? |
| Yes         No |
| We are planning to conduct interviews to understand the nursing faculty's perception of leaving academia. If you are interested, kindly write your email and contact number to schedule a time for the interview  Email __________________  Contact number _________________ |

1. ***Demographic Data***

| What is your gender? |
| --- |
| o             Male |
| o             Female |
| o             don’t want to state |
| Marital Status |
| o             Single |
| o             Married |
| o             Divorced |
| o             Widow |
| Number of children |
| o             No children |
| o             One child |
| o             Two children |
| o             Three children |
| o             More than three children |
| What is your age? |
|  |
| What is your educational background (Degree)? |
| o             Bachelor |
| o             Master |
| o             PhD |
| o             post-doctoral |
| What is your university type? |
| o             Public |
| o             Private |
| What is your job title? |
| o             Professor |
| o             Associate professor |
| o             Assistance professor |
| o             Lecturer |
| o             Adjunct faculty |
| o             Teaching assistant |
| How many years of experience do you have as a nursing faculty member? |
| o             Less than 1 year |
| o             1 to 2 years |
| o             3-5 years |
| o             6-8 Years |
| o             9-11 years |
| o             More than 11 Years |
| Are you full time faculty member? |
| o             Yes |
| o             No |
| What is your nationality? |

1. ***Interview questions***:

| 1. What was your path to academia? |
| --- |
| 2.  Can you tell me about your perceptions of leaving academia? |
| 3.  What are the Reasons that make you think of leaving academia? why |
| 4. What do you suggest as strategies to prevent/reduce rate of faculty member leaving academia ? |
